# Responses of belowground carbon allocation dynamics to extended shading in mountain grassland

**DOI:** 10.1111/nph.12138

**Published:** 2013-02-06

**Authors:** Michael Bahn, Fernando A Lattanzi, Roland Hasibeder, Birgit Wild, Marianne Koranda, Valentina Danese, Nicolas Brüggemann, Michael Schmitt, Rolf Siegwolf, Andreas Richter

**Affiliations:** 1 Institute of Ecology, University of InnsbruckSternwartestr. 15, 6020, Innsbruck, Austria; 2 Technische Universität München, Lehrstuhl für GrünlandlehreAlte Akademie 12, D-85350, Freising-Weihenstephan, Germany; 3 Department of Terrestrial Ecosystem Research, Faculty of Life Sciences, University of ViennaAlthanstrasse 14, A-1090, Vienna, Austria; 4 Forschungszentrum Jülich GmbH, Institute of Bio- and Geosciences, Agrosphere Institute (IBG-3)Leo-Brandt-Straße, 52425, Jülich, Germany; 5 Paul Scherrer Institute5232, Villigen PSI, Switzerland

**Keywords:** ^13^C tracer experiment, carbohydrate pools, carbon allocation, microbial phospholipid fatty acid (PLFA), respiration, starch, sugar

## Abstract

- Carbon (C) allocation strongly influences plant and soil processes. Short-term C allocation dynamics in ecosystems and their responses to environmental changes are still poorly understood.
- Using *in situ* ^13^CO_2_ pulse labeling, we studied the effects of 1 wk of shading on the transfer of recent photoassimilates between sugars and starch of above- and belowground plant organs and to soil microbial communities of a mountain meadow.
- C allocation to roots and microbial communities was rapid. Shading strongly reduced sucrose and starch concentrations in shoots, but not roots, and affected tracer dynamics in sucrose and starch of shoots, but not roots: recent C was slowly incorporated into root starch irrespective of the shading treatment. Shading reduced leaf respiration more strongly than root respiration. It caused no reduction in the amount of ^13^C incorporated into fungi and Gram-negative bacteria, but increased its residence time.
- These findings suggest that, under interrupted C supply, belowground C allocation (as reflected by the amount of tracer allocated to root starch, soil microbial communities and belowground respiration) was maintained at the expense of aboveground C status, and that C source strength may affect the turnover of recent plant-derived C in soil microbial communities.

## Introduction

Plants allocate photoassimilated carbon (C) to above- and belowground organs to fuel their metabolism, to provide C skeletons for growth and to build up storage pools (Larcher, 2003). A considerable amount of plant-assimilated C is also transferred to mycorrhizal symbionts, fungal endophytes and the rhizosphere, where exuded energy-rich compounds can stimulate nutrient mineralization in the soil and facilitate plant nutrient uptake (Lambers *et al*., 2008; Jones *et al*., 2009).

Plant C allocation has been suggested to be strongly sink driven, photosynthates being preferentially transferred to tissues with the highest demand (Lambers *et al*., 2008). Thus, under light limitation, plants tend to allocate a higher proportion of assimilated C to aboveground organs, whereas, under reduced nutrient and/or water supply, they invest more C to the root system (‘functional equilibrium hypothesis’, e.g. Bloom *et al*., 1985; Kobe *et al*., 2010; Poorter *et al*., 2012). However, it has been argued that the observed C allocation patterns could also be the result of a more complex suite of processes: in addition to sink strength, the C source could have a shared effect (Farrar & Jones, 2000), and C storage need not be purely passive, but could also be an active process, operating at the expense of growth (Chapin *et al*., 1990; Sala *et al*., 2012). In addition, with regard to C allocation to the rhizosphere, it is not clear to what degree it is driven by the plant (source) or mycorrhizal communities (sink) (Wright *et al*., 1998; Grimoldi *et al*., 2006; Jones *et al*., 2009; Lendenmann *et al*., 2011).

C allocation has been well studied with regard to carbohydrate metabolism (e.g. Smith & Stitt, 2007; Zeeman *et al*., 2007; Gibon *et al*., 2009; Graf & Smith, 2011; Werner & Gessler, 2011) and biomass partitioning (Poorter *et al*., 2012) of (often young) individual plants grown under controlled conditions, and as broader long-term patterns in ecosystems (Litton *et al*., 2007). Much less is known about the short-term dynamics of C allocation in ecosystems and how it responds to changing environmental conditions (Brüggemann *et al*., 2011; Epron *et al*., 2012). An analysis of the dynamic response of C transfer between different carbohydrate pools in above- and belowground plant organs, and between plants and microbial communities (Paterson *et al*., 2009), would improve our understanding of the processes underlying C allocation patterns in ecosystems.

Isotopic tracer studies have revealed a rapid and close coupling between photosynthesis and belowground C allocation to roots, soil organisms and respiratory processes in forests and grasslands (e.g. Ostle *et al*., 2000; Johnson *et al*., 2002; Leake *et al*., 2006; Denef *et al*., 2007; Högberg *et al*., 2008, 2010; Bahn *et al*., 2009; Epron *et al*., 2011). Only few studies have analyzed how such allocation dynamics are affected by changing environmental conditions. Reductions in light, nutrient or water supply have been shown to generally delay and reduce the release of recently assimilated C in soil or root respiration (Bahn *et al*., 2009; Rühr *et al*., 2009; Lehmeier *et al*., 2010; Barthel *et al*., 2011). It is largely unclear how such effects are related to changes in plant carbohydrate pools, although there is evidence in model plants that the respiratory substrate supply system may be very flexible, involving adjustments in the proportion of storage and turnover of current assimilates (Smith & Stitt, 2007; Lehmeier *et al*., 2010). Allocation to (and mobilization from) C storage pools, in particular, is poorly understood. In addition, the transfer of photosynthetic C to the rhizosphere may be affected by changing resource supply, and has been shown to decrease with fertilization in grassland (Denef *et al*., 2009). Direct effects of altered supply of photoassimilates (as, for example, induced by shading) on the dynamics of plant–microbe C allocation have so far not been studied in an ecosystem.

Here, we assess the importance of C storage in above- and belowground plant organs in grassland, and address the question of whether assimilate supply affects the dynamics of C allocation in the plant–soil system. Specifically, we test the hypothesis that shading (i.e. reduced C source strength) affects the dynamics of recently assimilated C in nonstructural carbohydrates and diminishes its transfer to roots and their storage pools, and to soil microbial communities. For this, canopy sections of a mountain grassland were pulse labeled with highly enriched ^13^CO_2_, and the fate of assimilated tracer was chased over a 1-month period in unshaded and shaded plots.

## Materials and Methods

### Site

The study was carried out on a mountain meadow at Kaserstattalm, Neustift, Austrian Central Alps, as described by Bahn *et al*. (2009). In brief, the meadow is fertilized with manure in spring every 2–4 yr, cut once in late July or early August and is lightly grazed in September. The dominating plant species are perennial and include the grasses *Anthoxanthum odoratum* L. and *Festuca rubra* L., and the forbs *Alchemilla vulgaris* L., *Leontodon helveticus* L.*, Leontodon hispidus* L. and *Trifolium repens* L. The soil is a dystric cambisol on siliceous bedrock with a topsoil pH of 5.5. The meadow is characterized by a comparatively high productivity and high soil respiration rates (Bahn *et al*., 2010; Schmitt *et al*., 2010). In the study year 2007, the meadow reached its peak biomass towards the end of July and was mowed in early August, immediately after the completion of the experiments. Peak above- and belowground biomasses of the site are in the range 240–440 and 420–980 g m^−2^, respectively (Bahn *et al*., 2006; Schmitt *et al*., 2010; M. Bahn *et al*., unpublished).

### Experimental set-up and treatments

In each of three consecutive campaigns during the period of peak biomass, we studied one experimental block containing a control and a shaded plot, as well as two plots which were pulse labeled and subsequently shaded or left unshaded. Labeling was performed during the late morning hours (starting between 08:45 and 11:20 h CET) on 15 and 26 July and on 1 August 2007. Microclimatic conditions during the experiments are shown in Supporting Information Fig. S1. Pulse labeling was accomplished by covering canopy sections with a transparent (95% light transmission) Perspex chamber of 1 × 1 × 0.7 m^3^ and continuously adding a small amount of 99.9 atom% ^13^C-CO_2_ to the chamber air, as described in Bahn *et al*. (2009). The chamber air temperature was stabilized by ice packs mounted on the back side of the chamber in the air flow. The frozen ice packs also prevented the condensation of water vapor on the chamber walls during measurements. During labeling, the temperature was maintained at 2°C near ambient, except for the last 20 min during the third labeling experiment, when the chamber air temperature reached values of up to 29°C, whereas ambient air temperature was near 22°C. The amount of ^13^CO_2_ added was manually regulated with a mass flow controller at average rates of *c*. 40 ml min^−1^ to keep the chamber CO_2_ mixing ratio at 600–800 ppmv throughout the labeling. This range was chosen to maximize photosynthetic uptake of the label, assuming that the short exposure of plants to elevated CO_2_ would not affect the allocation and respiratory use of C in the plant–soil system (Bahn *et al*., 2009).

Shading of unlabeled and labeled plots was achieved with tents of 3 × 3 m^2^ ground area and 2 m height. The tents were covered with nontransparent plastic sheets. Small slots at the bottom of the tent and the four corners facilitated an exchange of air. In the centre of the tents, where the plots were located, 5–8% of the incoming photosynthetically active radiation (PAR) was incident on the vegetation, as measured with a PAR sensor (SunScan SS1, Delta-T, Cambridge, UK) during the course of a sunny day. Shading treatments were started 1 h after the pulse labeling had been completed and lasted for 6–8 d.

### Sampling

Within each labeled and unlabeled plot, above- and belowground plant biomass and soil were sampled 2 (around noon; immediately before shading), 6, 12 and 34 h, 3.5 d, 1 wk (at the end of the shading treatment) and 1 month after the labeling had started. At each sampling, two 5 × 7-cm^2^ blocks of aboveground biomass and soil (10 cm depth, i.e. the main rooting horizon), located at the opposite ends of each plot, were harvested and mixed to obtain a pooled sample. Roots were separated from soil using tweezers, and root-free soil was immediately frozen using dry ice and stored in the laboratory at −20°C. Above- and belowground plant parts were killed and pre-dried by exposure to 1–2 min in a microwave, and oven dried to weight constancy on return to the laboratory. Aboveground biomass (subsequently termed ‘shoots’) included both leaves and stems, and belowground biomass (subsequently termed ‘roots’) comprised mostly fine roots and a minor amount of coarse roots, rhizomes and stolons.

### Analysis of plant and microbial compounds and their C isotope composition

#### Isotopic analysis of above- and belowground plant material

After drying, the collected samples from above- and belowground plant components were ground to a fine powder with a steel ball mill (MM 200, Retsch, Haan, Germany). Aliquots of the powdered samples of 0.5–0.8 mg were weighed into tin capsules (Säntis Analytical AG, Teufen, Switzerland) and placed into an autosampler AS-128 and injected into an elemental analyzer (EA-1110 CHN, both Carlo Erba, Milan, Italy). Under excess oxygen, the samples were combusted. The resulting CO_2_ was transferred in helium carrier gas via a variable open split interface (Conflo II) to a sector mass spectrometer (Delta S, both Finnigan MAT, Bremen, Germany) for the determination of the isotope ratio.

#### Analysis of carbohydrates

Plant material was dried and ground in a ball mill before further processing. For the analysis of sucrose, 30 mg of plant material was extracted with 1.5 ml of deionized water at 85°C for 30 min. Samples were centrifuged and the supernatant was transferred to ion-exchange cartridges (OnGuard II H and A 1-cm^3^ cartridges; Dionex, Thermo Scientific, Vienna, Austria) to remove ionic components. The neutral fraction was analyzed by high-performance liquid chromatography-isotope ratio mass spectrometry (HPLC-IRMS) (for a description of the system, see Wild *et al*., 2010) on a HyperREZ XP Ca^2+^ column (Thermo Scientific) at 85°C with 0.5 ml min^−1^ of deionized water as eluent. Standards of glucose and sucrose at a range of concentrations were measured, interspersed with the samples, to calculate sucrose concentrations and to correct for offsets in sucrose ^13^C values during analysis (Wild *et al*., 2010). For the analysis of starch, 100 mg of plant material was digested with α-amylase (Göttlicher *et al*., 2006; Richter *et al*., 2009), and the resulting glucose was measured by elemental analysis-isotope ratio mass spectrometry (EA-IRMS) (EA 1110, CE Instruments, and Delta Plus IRMS, Thermo Scientific). In the following, we limit our presentation of sugars to that of sucrose for the following reasons: the concentration of glucose was clearly lower than that of sucrose (*c*. 10–20% of sucrose); glucose was only slightly labeled (< 10% of the label in sucrose); and changes in the concentrations of glucose followed broadly those of sucrose. A representative number of above- and belowground samples were also analyzed for fructan (high-molecular-mass but water-soluble carbohydrate) by high-performance liquid chromatography-pulsed amperometric detection (HPLC-PAD) on a CarboPack PA-100 column (Dionex Corporation) to exclude the possibility that a substantial amount of carbohydrate remains undetected, but no significant fructan concentrations could be found.

#### Analysis of phospholipid fatty acids (PLFAs)

PLFAs were extracted from frozen soil samples by a mixture of methanol, chloroform and citrate buffer (2 : 1 : 0.8, v/v/v), and then separated from neutral lipids on silica columns, and finally subjected to alkaline methanolysis, as described in detail by Koranda *et al*. (2011). Dried fatty acid methyl esters (FAMEs) were re-dissolved in isooctane, and the concentrations and C isotope ratios of PLFAs were determined by a Trace Ultra GC (Thermo Scientific) interfaced with an IRMS (Delta V Advantage, Thermo Scientific) via a combustion unit (GC combustion II/TC, Thermo Scientific). A mixture of FAMEs (Supelco; Sigma-Aldrich) was used as a qualitative standard. An internal standard (19 : 0) was used for the calculation of FAME concentrations, as well as for the correction of δ^13^C values. δ^13^C values of PLFAs were corrected for the δ^13^C values of C added during methanolysis. We used the sum of fatty acids i15:0, a15:0, i16:0, i17:0, a17:0 as an indicator of Gram-positive bacteria and the sum of fatty acids 16:1ω9, 16:1ω7, 18:1ω7, 18:1ω5, cy17:0, cy19:0, cy18:0 as an indicator of Gram-negative bacteria. PLFAs 16:1ω5 and 20:4ω6 were used as markers for arbuscular mycorrhizal fungi (AMF); 18:1ω9 and 18:2ω6 were used as general fungal markers (Denef *et al*., 2009; Kaiser *et al*., 2010a,b).

#### Calculation and expression of C isotope composition

For all plant samples, we expressed the results as the ^13^C atom% excess, which corresponds to the increase in ^13^C atoms caused by pulse labeling expressed as the percentage of total C atoms present in each sample, and was calculated as the difference between the ^13^C atom% of the labeled and unlabeled samples. For PLFA samples, we expressed the results as the amount of ^13^C incorporated, which was calculated by multiplying the ^13^C atom% excess by the amount of PLFA in each sample.

### Measurements of leaf and root respiration

Leaf respiration was measured during early night-time (i.e. between 21:00 and 00:00 h) on five fully developed leaves of the dominant forb *Leontodon helveticus*, using a portable photosynthesis system (Li-6400, Li-Cor, Lincoln, NE, USA). Measurements were taken at the prevailing temperature of 15°C. Root respiration was measured during the daytime, as described by Bahn *et al*. (2006). At each sampling, six soil cores were extracted for each treatment, roots were carefully washed and fine roots (0–2 mm) were immediately measured in the field using a battery-operated gas exchange system (PLC-C connected to CIRAS-1, PPSystems, Hitchin, Hertfordshire, UK) (Bahn *et al*., 2006). The target temperature of 10°C, which corresponded to the soil temperature prevailing at the onset of the experiments, was achieved and stabilized using cooling boxes, in which the cuvette was placed.

### Data analysis

The effects of the shading treatment on the time courses of carbohydrate concentrations and plant and microbial ^13^C atom% excess and incorporated ^13^C were tested using repeated-measures ANOVA with sampling time as repeat, treatment as fixed factor and plot as random factor. To meet uncertainties concerning the normal distribution of the data caused by low replication, treatment effects per sampling time were analyzed using nonparametric tests. For carbohydrate concentrations with four to six replicates per sampling time and treatment, we applied Mann–Whitney *U*-tests; on isotope data with two to three replicates, we performed exact permutation tests.

## Results

### Plant tracer dynamics in unshaded plots

**Fig. 1:**
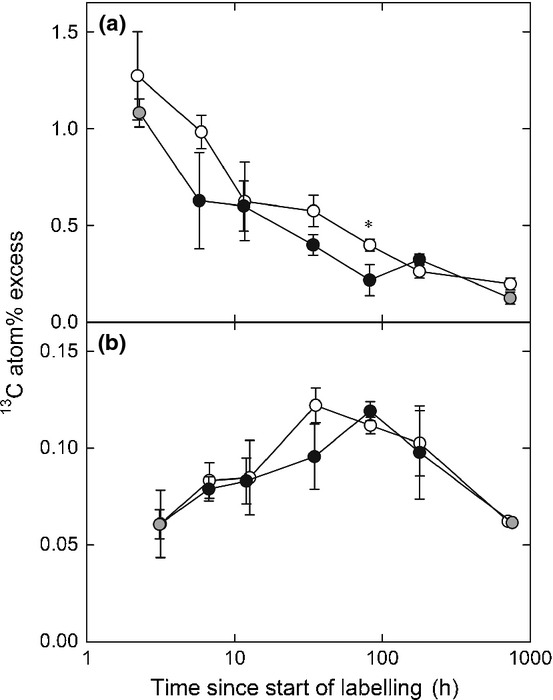
Time course of ^13^C atom% excess in (a) aboveground and (b) belowground plant biomass in unshaded (white circles) and shaded (black circles; gray circles indicate pre- and post-shading values) plots of a mountain meadow. *, Significant (*P* < 0.05) treatment effects for individual sampling dates.

**Fig. 2:**
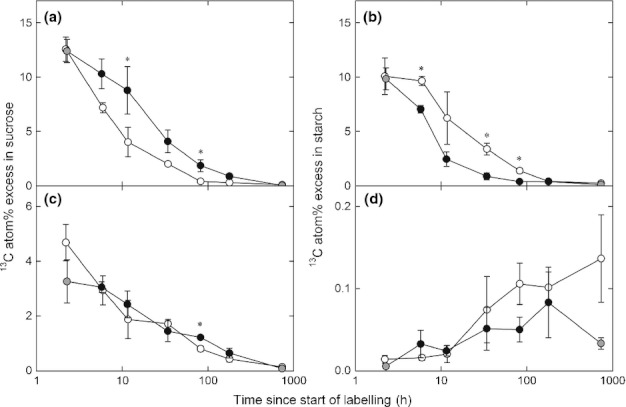
^13^C atom% excess in (a, c) sucrose and (b, d) starch of (a, b) aboveground and (c, d) belowground plant biomass in unshaded (white circles) and shaded (black circles; gray circles indicate pre- and post-shading values) plots of a mountain meadow. Error bars signify ± SE. *, Significant (*P* < 0.05) treatment effects for individual sampling dates.

At the first sampling, 2 h after the start of labeling, shoots and roots were both highly enriched in ^13^C, which reflects a rapid photosynthetic uptake and belowground translocation of labeled C (Fig. 1). This tracer signal was particularly pronounced for shoot sucrose and starch, and for root sucrose (Fig. 2a–c), which all showed similar tracer dynamics: following a rapid initial increase in ^13^C to values of 10–12.5 and 4.7 ^13^C atom% excess in shoots and roots, respectively, the tracer content declined over time, amounting to < 0.4 ^13^C atom% excess 1 wk after the pulse (Fig. 2a–c). By contrast, root starch continuously accumulated tracer at low rates, exhibiting the highest values (0.14 ^13^C atom% excess) 1 month after the labeling pulse (Fig. 2d).

### Shading effects on plant carbohydrate concentrations, tracer dynamics and allocation

**Fig. 3:**
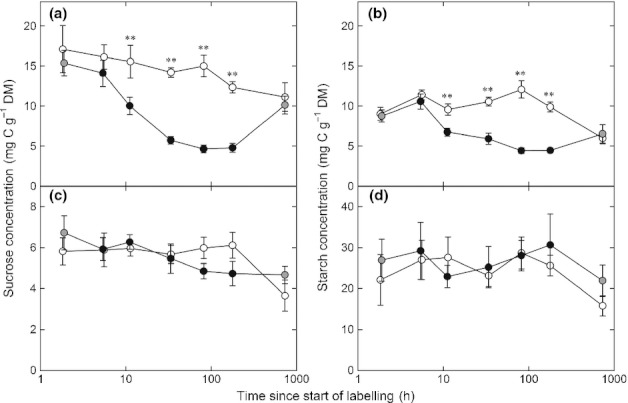
(a, c) Sucrose and (b, d) starch concentrations in (a, b) aboveground and (c, d) belowground plant biomass in unshaded (white circles) and shaded (black circles; gray circles indicate pre- and post-shading values) plots of a mountain meadow. Error bars signify ± SE. **, Significant (*P* < 0.01) treatment effects for individual sampling dates. Note that the first sampling was made around noon, the second in the afternoon, and the subsequent samples were taken at night.

At the beginning of the experiments, mean shoot and root sucrose concentrations pooled across all plots were 16.2 and 6.3 mg g^−1^, respectively, and shoot and root starch concentrations were 8.9 and 24.5 mg g^−1^, respectively. Shoot sucrose and starch concentrations were reduced significantly by shading within half a day and, after 4–8 d of shading, amounted to only 31–39% (sucrose) and 37–45% (starch) of the values in control plots (Fig. 3, Table 1). By contrast, root sucrose and starch concentrations were not reduced significantly by shading (Fig. 3, Table 1). Three weeks after the shading experiment had been completed, shoot and root sucrose and starch concentrations showed no difference between previously shaded plots and control plots, and were generally lower than the values at the start of the experiment.

**Table 1:** Results of repeated-measures ANOVA to test for overall effects of treatment, time and time–treatment interactions in plant carbohydrate concentrations, ^13^C atom% excess of plant biomass and carbohydrates and incorporated ^13^C of soil microbial phospholipid fatty acids (PLFAs) in the mountain meadow

	Treatment		Time		Time × treatment	
			
	*F* value	*P* value	*F* value	*P* value	*F* value	*P* value
Sucrose concentration in shoots	103.83	**< 0.001**	0.30	0.874	6.13	**0.001**
Sucrose concentration in roots	1.14	0.313	1.16	0.345	1.06	0.391
Starch concentration in shoots	64.03	**< 0.001**^a^	5.20	**0.002**	10.13	**< 0.001**
Starch concentration in roots	0.05	0.834^a^	1.08	0.383	0.141	0.966
^13^C atom% excess in shoot biomass	1.05	0.382	0.11	0.873^b^	0.68	0.526^b^
^13^C atom% excess in root biomass	0.03	0.865	2.98	0.105^b^	0.61	0.605^b^
^13^C atom% excess in shoot sucrose	6.90	0.079	7.97	**0.041**^b^	1.53	0.299^b^
^13^C atom% excess in root sucrose	0.23	0.668	2.64	0.182^b^	0.61	0.527^b^
^13^C atom% excess in shoot starch	7.08	0.076^a^	3.97	0.135^b^	1.41	0.321^b^
^13^C atom% excess in root starch	0.42	0.563^a^	0.53	0.623^b^	0.69	0.543^b^
^13^C atom% excess in AMF PLFAs^c^	31.23	0.113	0.97	0.506	1.31	0.457
^13^C atom% excess in fungal PLFAs^c^	11.32	0.184-	2.35	0.368^b^	1.96	0.395^b^
^13^C atom% excess in Gram-positive bacterial PLFAs	0.70	0.557	10.28	0.193^b^	1.49	0.438^b^
^13^C atom% excess in Gram-negative bacterial PLFAs	2947.86	**0.012**	0.64	0.571^b^	1.19	0.473^b^
^13^C atom% excess in all measured PLFAs^c^	36.96	0.104	2.22	0.376^b^	1.30	0.458^b^
Leaf respiration rate	2.58	0.129	6.69	**0.001**	8.07	**< 0.001**
Root respiration rate	4.67	0.059^a^	3.91	**0.01**	1.11	0.368

Pre- and post-treatment data were excluded from the analysis. Bold values highlight significant effects at *P* < 0.05.

a Violation of homoscedasticity in at least one dataset (*P* value of Levene test < 0.05).

b Glasshouse-Geisser correction was applied because of violation of sphericity (*P* value of Mauchly test < 0.05).

c Dataset of last sampling time was excluded to obtain consistent time series across all samplings.

**Fig. 4:**
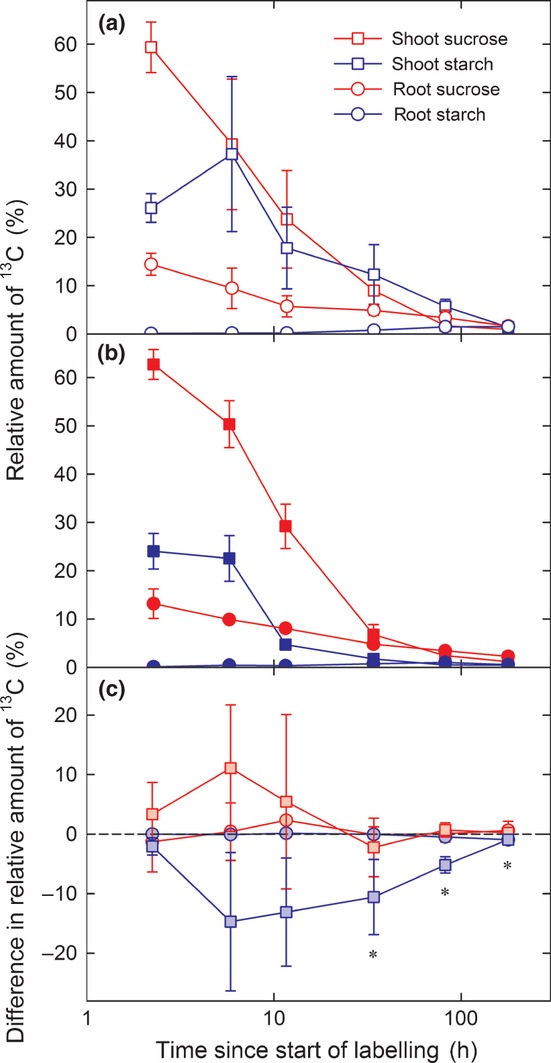
Relative amount of tracer ^13^C pools in shoot and root sucrose and starch in (a) unshaded (open symbols) and (b) shaded (closed symbols) plots of a mountain meadow, and (c) the difference in the respective relative pool sizes of shaded minus unshaded plots. The amounts of ^13^C in the respective pools are expressed as a fraction (%) of the total amount of ^13^C recovered in all of these carbohydrate pools at the first sampling (2 h after the start of labeling and immediately before the start of the shading experiment). Error bars signify ± SE. *, Values for shoot starch are significantly (*P* < 0.05) different from zero. The partitioning of pools is based on the assumption that biomass and root/shoot ratios remained constant during the week of shading.

Shading reduced the initial rate of decline in tracer content in shoot biomass (Fig. 1) and in shoot and root sucrose (Fig. 2a,c). It accelerated the initial decline in tracer content in shoot starch, causing consistently lower values of ^13^C excess during the first 3 d of shading (Fig. 2b). This was clearly reflected in the allocation dynamics of shoot carbohydrate pools (Fig. 4), indicating that the ^13^C pool in starch decreased by 15% within 3–4 h after shading, whereas the ^13^C pool in sucrose increased by 11% (Fig. 4b). The difference in tracer contents and pools in shoot sucrose and starch between shaded and unshaded plots disappeared largely after 1 wk of shading (Figs 2a,b, 4). In root starch, tracer contents increased more slowly in shaded relative to control plots (Fig. 2d), and accumulated a somewhat smaller fraction of assimilated tracer relative to controls. The relative amount of ^13^C allocated to the root starch pool was generally small (0.8–1.8% of the initial amount of ^13^C recovered in the measured carbohydrates; Fig. 4). Three weeks after the end of the shading experiment, tracer concentrations did not differ significantly between previously shaded and unshaded plots, except for root starch, which showed lower tracer concentrations in previously shaded plots (Fig. 2a–d), although the differences were not significant.

### Shading effects on leaf and root respiration

**Fig. 5:**
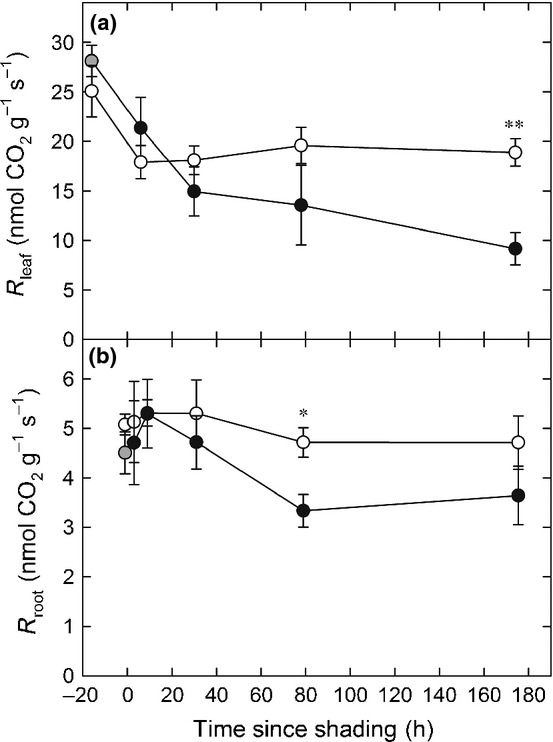
(a) Specific (mass-based) night-time leaf respiration (at 15°C) of the dominant forb *Leontodon helveticus* and (b) specific fine root respiration (at 10°C) in unshaded (white circles) and shaded (black circles; gray circles indicate pre-shading values) plots of a mountain meadow. Error bars signify ± SE. * and **, Significant treatment effects for individual sampling dates at the levels of *P* < 0.05 and *P* < 0.001, respectively.

Shading caused a significant reduction in night-time leaf respiration rates at the reference temperature: by the end of the shading experiment, leaf respiration of *Leontodon helveticus* amounted to < 50% in shaded plots relative to control plots (Fig. 5a), when expressed on a dry weight basis. As a result of a significant (*P* < 0.001) decrease in leaf mass per area (LMA) from 33.2 to 26.7 g m^−2^ under shading, leaf respiration per unit leaf area was reduced to < 30% of the values measured in control plots. Shading had a less pronounced effect on specific root respiration at the reference temperature: after 1 wk of shading, it was reduced to 77% of the values observed in control plots (Fig. 5b).

### C transfer to microbial communities in unshaded and shaded plots

**Fig. 6:**
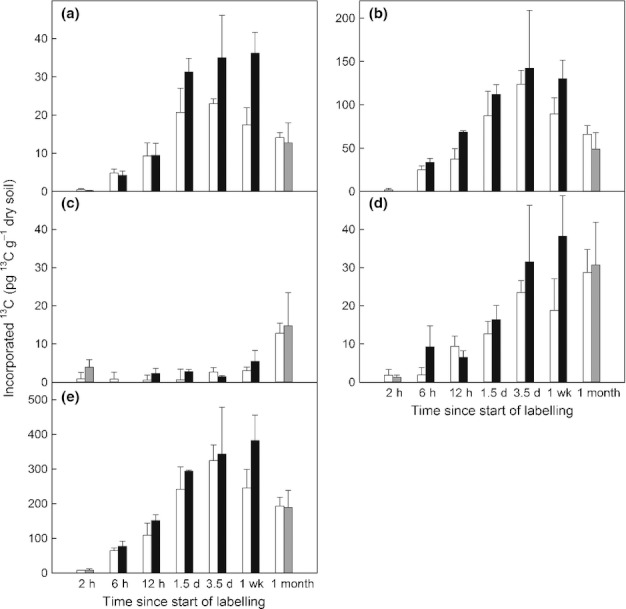
Time course of the amount of ^13^C incorporated into phospholipid fatty acids (PLFAs) of microbial groups in unshaded (white bars) and shaded (black bars; gray bars indicate pre- and post-shading values) plots of a mountain meadow. (a) Arbuscular mycorrhizal fungal (AMF) PLFAs (16:1ω5, 20:4ω6), (b) general fungal PLFAs (18:1ω9c, 18:2ω6,9c), (c) Gram-positive bacterial PLFAs (i15:0, a15:0, i16:0, i17:0, a17:0), (d) Gram-negative bacterial PLFAs (18:1ω7, cy18:0(11/12), 16:1ω9, cyc17:0), (e) all PLFAs (including also general bacterial biomarkers). Error bars signify + SE. For individual sampling dates, treatment effects were not significant at *P* < 0.05.

Four hours after the labeling had been completed, a first distinct tracer signal was detected in the PLFAs of fungal communities and Gram-negative bacteria (Fig. 6). For these microbial groups, the amount of ^13^C incorporated in the PLFAs of control plots peaked 2 d after labeling. One month after labeling, tracer content was reduced significantly in the fungal PLFAs, but had increased further in the PLFAs of Gram-negative bacteria (Fig. 6). During the first days after labeling, Gram-positive bacteria incorporated only negligible amounts of ^13^C into their PLFAs, and showed a significant tracer signal after 1 month.

Shading had no significant effect on ^13^C tracer content or dynamics (except for Gram-negative bacteria, Table 1); however, the peak values observed for PLFAs of AMF and Gram-negative bacteria tended to occur somewhat later, so that, at the end of the shading treatment (Fig. 6, 1 wk), slightly more ^13^C was incorporated relative to PLFAs in control plots, but the differences were not significant. Three weeks after the shading experiment ended, the amount of tracer in PLFAs of all microbial groups was similar for unshaded and previously shaded plots (Fig. 6). In general, no relationship between ^13^C in root sucrose and in PLFAs was found in unshaded and shaded plots (not shown).

## Discussion

C allocation is a major process underlying the patterns of plant growth and biomass partitioning, rhizosphere processes and the coupling of photosynthesis and respiration in plants and terrestrial ecosystems (Brüggemann *et al*., 2011; Poorter *et al*., 2012). Unfortunately, our understanding of the multiple pathways and controls underlying C allocation is still rather limited, although progress has been made in model plants with regard to the role and controls of carbohydrate metabolism and its links to respiration and growth (Smith & Stitt, 2007; Zeeman *et al*., 2007; Graf & Smith, 2011). At the ecosystem scale, the assessment of pathways and responses of C allocation to environmental changes is particularly challenging, because of the large numbers of species and their interactions that typically constitute natural and semi-natural ecosystems, the fluctuating environmental conditions and spatial variability, and the technical limitations which complicate frequent *in situ* monitoring of the complete set of C fluxes between different ecosystem pools and compartments.

We assessed belowground C allocation in grassland and its response to extended shading by pulse labeling canopy sections with highly enriched ^13^CO_2_ and measuring the temporal dynamics of five complementary sets of parameters: (1) the ^13^C content in plant biomass and root and shoot sucrose and starch pools; (2) root and shoot sucrose and starch concentrations; (3) leaf and root respiration at the respective reference temperatures; (4) the ^13^C content in PLFAs of microbial groups; and (5) the ^13^C content and amount of soil-respired CO_2_ (published in Bahn *et al*., 2009). Isotopic pulse labeling permits an assessment of the allocation dynamics of C of a well-defined age (as assimilated during the short labeling period). However, it does not indicate the total amount of C being allocated in the system. Thus, temporal changes in sucrose and starch concentrations provide important complementary information on the allocation dynamics of the total nonstructural C pool. In addition, leaf and root respiration rates at reference temperatures indicate, to what extent, reductions in respiratory substrate pools under shading limit metabolic processes.

### Tracer dynamics in plant carbohydrate pools

Tracer dynamics showed that the C assimilated during labeling was rapidly incorporated into shoot sucrose and starch pools and transferred below ground, a very distinct tracer signal appearing in root sucrose and, to a lesser extent, in starch within 2 h after labeling had started (Fig. 2). The tracer uptake and translocation were so rapid and substantial that they were also immediately evident in the total plant biomass (Fig. 1) and in belowground respiration (Bahn *et al*., 2009). Tracer dynamics in shoot starch were consistent with the notion that, during the daytime, a significant amount of newly assimilated C is deposited in starch and mobilized in the subsequent night (Zeeman *et al*., 2007; Graf & Smith, 2011): from the first (noon) to the second (afternoon) sampling, the amount of ^13^C in starch pools increased, but decreased sharply at night (Fig. 4a). C mobilized from starch at night has been suggested to be an important substrate for respiration and growth both above- and belowground (Smith & Stitt, 2007; Lehmeier *et al*., 2008; Barthel *et al*., 2011). Accordingly, the diurnal patterns in C isotope composition of soil-respired CO_2_ observed after labeling (Bahn *et al*., 2009) could be related to such diurnal patterns of transitory starch accumulation and remobilization. In contrast with shoot starch, root starch did not exhibit a reduction in tracer concentration during the week of the experiment, but continuously accumulated a small amount of ^13^C (Fig. 4a). This could indicate that root starch acts as a seasonal store, which is also supported by a higher starch-to-sucrose ratio in roots relative to shoots.

Shading triggered a rapid and extensive mobilization of C stores in shoot starch: shoot starch rapidly lost ^13^C, whereas shoot sucrose transiently increased its ^13^C pool (Fig. 4). By contrast, shading did not alter the pattern of tracer accumulation in root starch (Fig. 4). The implications are discussed further below (section ‘Effects of extended shading on C allocation’).

### C transfer to microbial communities of unshaded and shaded plots

Our study has demonstrated a rapid transfer of fresh photoassimilates from leaves to microbial communities and a significant incorporation in PLFAs within a few hours, with peak values occurring after 1–2 d. This is in support of earlier grassland studies (Treonis *et al*., 2004; Leake *et al*., 2006; Denef *et al*., 2007; De Deyn *et al*., 2011), whereas, for forests, peak values of tracer recovery in microbial biomass have been reported after 3–6 d (Högberg *et al*., 2010; Epron *et al*., 2011).

The patterns of ^13^C incorporation by different microbial groups observed in our study are consistent with observations in earlier grassland studies: fungi (including AMF) and Gram-negative bacteria incorporate the tracer rapidly, whereas Gram-positive bacteria are characterized by a delayed uptake of labeled C. The rapid uptake of plant-derived C by fungi has been documented previously (Johnson *et al*., 2002; Treonis *et al*., 2004; Olsson & Johnson, 2005; Denef *et al*., 2007; Drigo *et al*., 2010; De Deyn *et al*., 2011) and is in line with the notion that AMF and other fungi have direct access to plant carbohydrates. Amongst nonmycorrhizal fungal groups, dark septate endophytes could have contributed to the tracer dynamics at our site, where they have been observed abundantly (M. Van der Heijden, pers. comm.). For Gram-negative bacteria, our study suggests a slightly faster ^13^C uptake than reported by Denef *et al*. (2007), demonstrating that, within < 12 h from the start of labeling, significant amounts of tracer were incorporated in their PLFAs. This suggests that Gram-negative bacteria were probably able to feed directly on root or fungal exudates, but they were also able to recycle C which had previously been incorporated in roots and/or other microbial groups, as indicated by peak values of ^13^C occurring 1 month after labeling.

Our results confirm previous observations that Gram-positive bacteria take up tracer more slowly than do fungi and Gram-negative bacteria (Olsson & Johnson, 2005; Denef *et al*., 2007), which has been attributed to an assimilation of C from fungal necromass or dead root material (Denef *et al*., 2007). This indicates that root exudates are only a minor source of C for the growth of Gram-positive bacteria, although they may potentially play an important role for an enhanced turnover of older soil organic matter by this microbial group through priming effects (Bird *et al*., 2011).

While the results for unshaded plots thus largely confirm and refine earlier findings on the close linkage between plants and microbial communities, our study provides novel insights as to how the supply of photoassimilates affects this linkage. Surprisingly, shading did not reduce the transfer of freshly assimilated C to soil microbial communities: neither the speed of transfer nor the amount of ^13^C incorporated in PLFAs was reduced by shading (Fig. 6). By contrast, the amount of recovered tracer tended to be higher in shaded plots. In unshaded plots, the amount of ^13^C incorporated in PLFAs of fungi and Gram-negative bacteria decreased after 2 d, whereas it remained constant (or increased slightly) under shading. It has been discussed previously that a decrease in tracer concentration in PLFAs could potentially be caused by a dilution of ^13^C by unlabeled fresh C via plant roots after pulse labeling (Denef *et al*., 2007; De Deyn *et al*., 2011; Balasooriya *et al*., 2012). To analyze tracer dynamics independently of such a possible dilution effect, we calculated the absolute amount of ^13^C contained in PLFAs (Fig. 6). The observed reductions in the amounts of ^13^C thus reflect solely the fact that the tracer previously incorporated in PLFAs was turned over (and subsequently respired, taken up by saprophytic organisms or remained in the soil solution). We conclude that the residence time of C in microbial groups characterized by a rapid incorporation of recent C (i.e. fungi and Gram-negative bacteria) is affected by assimilate supply. Possible mechanisms could be an increased lifetime (reduced turnover) under conditions of decreased C input and increased recycling of C contained in these microbial groups (i.e. a shift in predominant C source), as well as a change in C use efficiency (Manzoni *et al*., 2012).

### Effects of extended shading on C allocation

It is commonly held that C allocation in plants is driven by the ‘demand’ and the ‘sink strength’ of different tissues/organs, although it is extremely difficult to quantify such parameters or to assess the relative role of source strength and of different sinks (Farrar & Jones, 2000; Poorter *et al*., 2012). We tested the response of C allocation dynamics to manipulations of the C source, and observed that sustained shading decreased carbohydrate pools above- but not belowground, and reduced leaf respiration more strongly than root respiration.

Notably, the response of leaf respiration to shading was delayed relative to that of carbohydrate pools, which is in line with an earlier study on alpine species, where, after a single day of shading, carbohydrates but not leaf respiration rates were consistently diminished (McCutchan & Monson, 2001). It has been suggested that, in response to extended shading, the pools of respiratory intermediates could be replenished by a switch to catabolism of proteins, cell walls and lipids (Brouquisse *et al*., 1998; Smith & Stitt, 2007). This might explain the observed partial decoupling of leaf carbohydrates and respiration rates. Root respiration was less affected by shading, which largely supports conclusions from a clipping experiment in the same grassland indicating that belowground C pools can buffer root metabolic activity over periods of at least 1–2 wk (Bahn *et al*., 2006). The observed reductions in root respiration might be explained by reduced C allocation to growth (and thus growth respiration) under extended darkness (Robson & Parsons, 1981; Smith & Stitt, 2007).

C allocation to storage has often been considered to be largely ‘passive’, resulting from an overflow of carbohydrates not used for respiration and growth (see review by Chapin *et al*., 1990). Interestingly, our results indicate that, even under severe C limitation, when aboveground carbohydrate pools were strongly depleted, leaf respiration was down-regulated and leaf mass per leaf area declined, recent C continued to be incorporated into belowground storage pools (i.e. starch). Such ‘active’ storage (Chapin *et al*., 1990) may play an underestimated role for the survival and growth under potentially C-limiting situations, as has been argued for trees only very recently (Sala *et al*., 2012; Wiley & Helliker, 2012). In mountain grassland, it could reflect an evolutionary adaptation to grazing, as well as the short growing season, which both require the presence of belowground storage pools for regrowth (Chapin *et al*., 1990; Donaghy & Fulkerson, 1998). By buffering against asynchrony between supply and C requirement of respiration and growth, ‘active’ storage is driven by a ‘demand’ extending beyond the immediate C requirements for maintenance and growth. Thus, even though the functional equilibrium hypothesis predicts a preferential allocation of C to aboveground organs in situations of severe light limitation, our results suggest that no major change in allocation occurred. More generally, it should be noted that C allocation to a storage pool is probably always the result of an active process, as, for example, reflected in the highly complex and dynamic relationships between sugar and starch pools between day and night (Smith & Stitt, 2007; Zeeman *et al*., 2007), and that therefore the expression ‘passive storage’ may need to be reconsidered altogether.

C allocation to soil microbial communities can be substantial, amounting to *c*. 5–20% of photosynthesis for AMF alone (Jakobsen & Rosendahl, 1990; Johnson *et al*., 2002; Grimoldi *et al*., 2006). It is unclear to what extent it is driven by the plant (source) or fungal/mycorrhizal communities (sink) (Grimoldi *et al*., 2006; Jones *et al*., 2009). There is evidence that C sink strength by AMF can stimulate rates of photosynthesis and thus C source strength (Wright *et al*., 1998; Kaschuk *et al*., 2009; Lendenmann *et al*., 2011). Our study showed that the amount of recent C incorporated into PLFAs of fungal (including AMF) and bacterial communities was not reduced by shading, that is, their C sink strength was not affected by reduced C source strength. This suggests that C allocation to mycorrhizae and the rhizosphere is strongly sink driven. However, it should also be noted that we did not quantify mycorrhizal (and rhizosphere) respiration, which may consume a large percentage of C allocated to AMF (Johnson *et al*., 2002; Grimoldi *et al*., 2006). Combining the findings that shading had only a minor effect on total soil respiration (Bahn *et al*., 2009) or root respiration (Fig. 5), and did not reduce the total amount of tracer recovered from soil-respired CO_2_ (Bahn *et al*., 2009; Fig. S2), we can nevertheless assume that the respiration (and its use of recent C) of AMF and other microbial communities was not reduced, and that therefore no reduction in overall rhizosphere sink strength occurred under reduced source strength.

### Conclusions

In the grassland studied, shoot and root starch played very different roles for C storage. Shoot starch accumulated recent C rapidly, and shading triggered an immediate mobilization of this C store. By contrast, root starch, which received comparatively little C, acted as a long-term storage, whose behavior remained unaffected by shading.

Notably, belowground C allocation was insensitive to a distinct reduction in C source, as the amount of tracer allocated to root starch, incorporated into soil microbial communities and lost in belowground respiration was similar in shaded and unshaded plots. This supports the hypothesis of a preferential C flow to belowground plant functions (respiration and storage), and to fungal communities and rhizosphere microbes, at the expense of the C status of aboveground organs. However, the altered patterns of tracer residence times under shading also suggest an influence of C source strength on the utilization and turnover of recent plant-derived C in soil microbial communities.

## Supporting Information

**Fig. S1** Photosynthetically active radiation, air and soil temperature, and soil water content during the study period.

**Fig. S2** Cumulative amount of tracer respired below ground in unshaded and shaded plots during the first and the second experiment.
