# Supplementary material for: Responses of belowground carbon allocation dynamics to extended shading in mountain grassland
Source: New Phytol. 2013 Feb 6;198(1):116–26. doi: 10.1111/nph.12138 (PMC3592993; doi:10.1111/nph.12138)
Supplement: Supplementary file 1 [file nph0198-0116-SD1.doc]

**Supporting Information Figs S1 & S2**


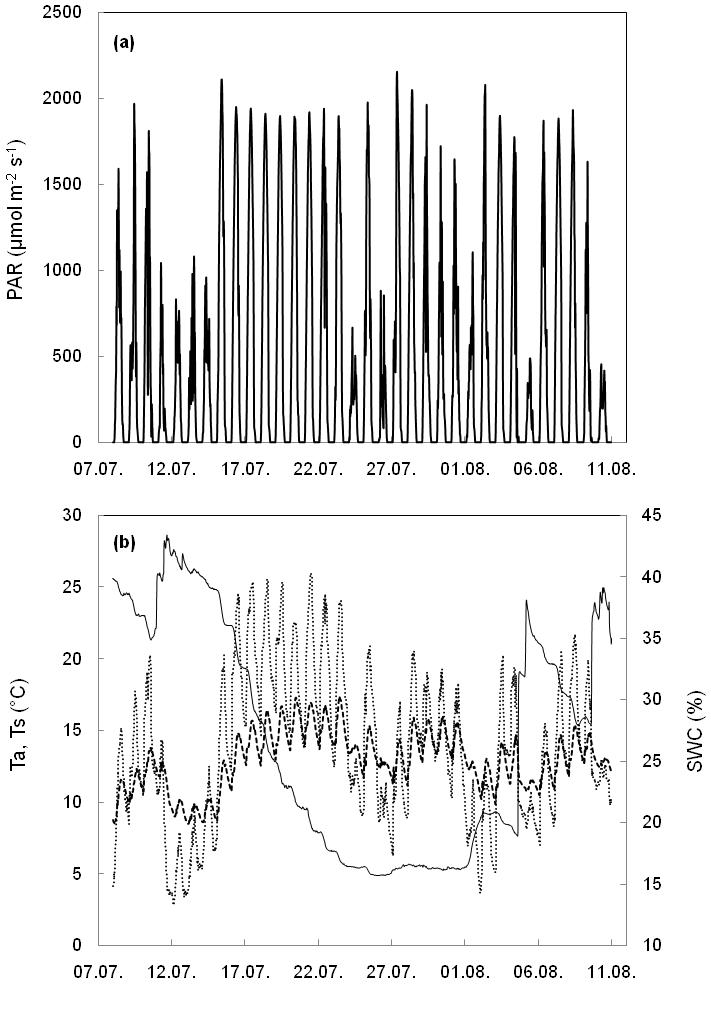


**Fig. S1** (a) Photosynthetically active radiation (PAR) and (b) air temperature (Ta; dotted line), soil temperature (Ts; dashed line) and soil water content (SWC; solid line) at 5 cm depth of a mountain meadow during the study period.

**Fig. S2** Cumulative amount of tracer (mg ^13^C) respired belowground in unshaded (dashed line) and shaded plots (solid line) of a mountain meadow during the first and the second experiment (data from Bahn *et al*., 2009). Error bars represent standard errors. The dataset for the third experiment was too short and incomplete for deriving defensible estimates of cumulative excess ^13^C respired. The data for the first experiment extended for some more days than displayed here and indicate that the shown trend in the cumulative amount of tracer recovered in unshaded and shaded plots persists.
